# Removal, Refixation, and Augmentation of a Broken Iliosacral Screw: A Percutaneous Surgical Technique

**DOI:** 10.1155/cro/5589320

**Published:** 2026-04-10

**Authors:** Nitesh Raj Pandey, Bishwa Bandhu Niraula, Prashanna Dip Karki, Ramesh Aryal, Bibek Banskota

**Affiliations:** ^1^ Department of Orthopaedics, Baidya & Banskota Hospital, Gwarko, Lalitpur, Nepal

**Keywords:** broken screw, fluoroscopy-guided surgery, iliosacral screw, pelvic ring injury, revision fixation

## Abstract

**Background:**

The management of a broken iliosacral screw in an anterior–posterior compression (APC)–type pelvic ring injury presents a surgical challenge, particularly in cases requiring removal and refixation without navigation assistance.

**Objective:**

This study describes a step‐by‐step surgical technique for safely removing a fractured S1 cannulated iliosacral screw and replacing it with a larger‐diameter screw, supplemented with additional fixation (e.g., S2 screw or iliosacral fixation) to enhance stability.

**Technique:**

The procedure involves careful fluoroscopy‐guided extraction of the broken screw, followed by reaming and placement of a larger screw for improved fixation. Supplementary stabilization (e.g., S2 screw or transiliac–transsacral fixation) is then added to reinforce the construct.

**Conclusion:**

This technique provides a reliable solution for managing broken iliosacral screws in APC pelvic injuries, even without navigation, while optimizing biomechanical stability through larger screw replacement and supplemental fixation.

## 1. Introduction

Pelvic ring injuries, particularly those resulting from high‐energy trauma such as anterior–posterior compression (APC) mechanisms, often require surgical stabilization to restore stability and facilitate functional recovery [1, 2]. Iliosacral screw fixation remains a widely used technique due to its biomechanical advantages and minimally invasive nature [3, 4]. However, complications such as screw breakage often attributed to excessive micromotion, delayed union, or suboptimal screw positioning pose significant challenges in revision surgery [5, 6].

The removal of a broken S1 iliosacral screw presents unique technical difficulties, especially in the absence of intraoperative navigation, which is not universally available in all trauma centers [7]. Broken screws within the dense sacral bone require precise extraction techniques to avoid further bone loss or iatrogenic neurovascular injury [8]. Furthermore, revision fixation demands biomechanically superior constructs, often necessitating a larger‐diameter screw and supplemental fixation (e.g., S2 iliosacral screw or transsacral–transiliac fixation) to enhance stability and reduce the risk of recurrent failure [9].

While several studies have discussed primary iliosacral screw fixation techniques, literature on salvage procedures for broken screws particularly in APC‐type injuries remains limited. This paper describes a step‐by‐step surgical technique for safely removing a fractured S1 iliosacral screw without navigation, replacing it with a larger screw, and augmenting stability with supplementary fixation. The approach emphasizes fluoroscopic guidance, meticulous screw extraction, and strategies to optimize revision construct strength. Leaving a broken iliosacral screw can lead to several potential complications such as risk of broken part migration, infection, future revision surgical challenges, persistent pain, and dysfunction.

## 2. Patient and Methods

We present the case of a 42‐year‐old female who sustained a pelvic ring injury following a road traffic accident. The injury was classified as an APC Type II pelvic ring injury.

Initial surgical management included posterior fixation using a 6.5 mm × 90 mm fully threaded cannulated S1 iliosacral screw on the right side, along with anterior pelvic stabilization using a 10‐hole plate with five screws.

The patient remained nonweight‐bearing for 2.5 months following surgery.

### 2.1. Medical History

The patient had no history of diabetes mellitus, hypertension, osteoporosis, or other chronic medical illnesses. She was a nonsmoker and had no history of long‐term steroid use or metabolic bone disease. At presentation, she was taking intermittent nonsteroidal anti‐inflammatory medication for pain relief but was otherwise not on any regular medication.

At 5 months postoperatively, routine radiographs demonstrated breakage of the iliosacral screw at the fifth thread from the screw head.

The patient reported persistent right posterior pelvic pain (VAS score 6/10), particularly during ambulation.

### 2.2. Diagnostic Assessment

Possible causes of the patient’s symptoms included the following:

• Pain due to hardware failure
• Pseudoarthrosis of the posterior pelvic ring
• Sacroiliac joint degeneration
• Mechanical irritation from the implant

**Figure 1:**
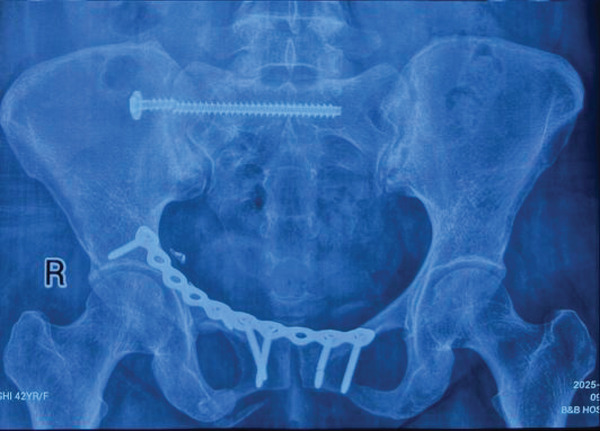
Prerevision AP pelvis radiograph showing a right S1 iliosacral screw broken at the level of the fifth thread from the screw head with the anterior plate in situ.

Radiographic evaluation showed a fracture of the S1 screw at the level of the fifth thread from the screw head without secondary displacement of the pelvic ring (Figure 1). The anterior plate remained intact, and there was no evidence of implant loosening.

A prerevision CT scan was not performed, as the position of the broken screw and the sacral corridor could be sufficiently assessed using standard pelvic radiographs combined with intraoperative fluoroscopic views (anteroposterior [AP], inlet, outlet, and lateral views).

### 2.3. Revision Surgery

Revision surgery was planned due to the following:

• Persistent pain
• Risk of mechanical instability
• Potential migration of the retained screw fragment

After discussion of the risks and benefits of revision surgery, the patient agreed to undergo hardware removal and revision fixation.

## 3. Timeline of Clinical Events

The timeline of clinical events is summarized in Table 1.

**Table 1:** Timeline of clinical events.

Time point	Event
Day 0	Road traffic accident ‐ APC II pelvic ring injury
Day 3	Primary fixation with an S1 iliosacral screw and anterior plating
2.5 months	Initiation of weight‐bearing
5 months	Broken iliosacral screw detected
6 months	Revision surgery performed
2 weeks postop (revision)	Wound review ‐ no neurological deficit
3 months postop (revision)	Stable fixation, pain improved
6 months postop (revision)	Full weight‐bearing without pain

## 4. Surgical Technique

The patient was placed supine on a radiolucent table under general anesthesia. The pelvis was positioned near the edge of the table to allow adequate C‐arm movement for AP, inlet, outlet, and lateral views.

Prophylactic intravenous cefuroxime (750 mg) was administered preoperatively.

A 1‐cm incision was made over the previous screw entry point. The screw head was identified and cleared of soft tissue using curettage.

**Figure 2:**
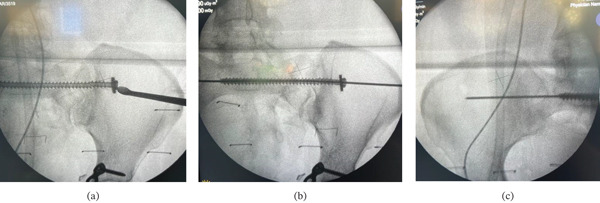
(a) Intraoperative identification and soft tissue clearance of the screw head using curettage. (b) A long guidewire was carefully advanced through the canal of the broken screw. (c) Final fluoroscopic confirmation demonstrating the guidewire’s successful traversal across the contralateral sacroiliac joint at the corresponding level.

A long guidewire was introduced through the canal of the broken screw. The guidewire successfully traversed the retained fragment and exited through the contralateral sacroiliac joint (Figure 2).

### 4.1. Removal of the Broken Screw Head

**Figure 3:**
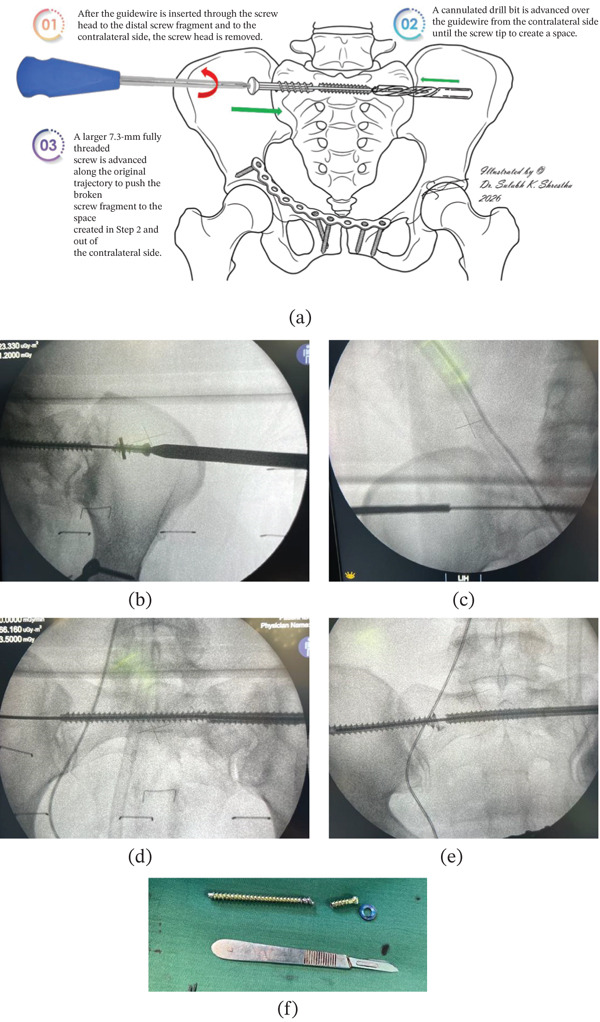
(a) Schematic illustration of the push‐through technique for removal of the broken iliosacral screw. (b) Removal of the broken screw head using a cannulated screwdriver system. (c) Progressive advancement of a cannulated drill along the contralateral end of the guidewire until reaching the distal tip point of the broken screw. (d) Insertion of a 7.3‐mm fully threaded cannulated screw from the right side following the same guidewire trajectory. (e) Progressive extrusion of the retained screw part toward the left iliac cortex during advancement of the new replacement screw. (f) Extracted broken screw fragments with the washer.

The proximal screw head was removed using a cannulated screwdriver. The washer was then removed through the same incision (Figure 3b).

### 4.2. Contralateral Access

A small stab incision was made over the left iliac crest along the trajectory of the guidewire. A cannulated drill was advanced over the guidewire up to the tip of the retained screw fragment to create space for extrusion (Figure 3c).

### 4.3. Push‐Through Technique

A 7.3 mm × 100 mm fully threaded cannulated screw was inserted along the original trajectory from the right side. As the screw advanced, it engaged the retained screw fragment and pushed it toward the contralateral side (Figure 3D,E).

With controlled advancement and gentle tapping, the retained fragment was extruded through the contralateral incision and removed using a surgical plier (Figure 3f).

### 4.4. Revision Fixation

The newly inserted 7.3‐mm screw provided improved fixation due to its larger diameter and longer length.

**Figure 4:**
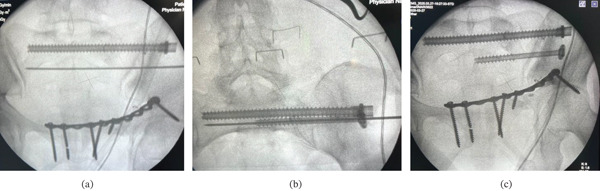
(a) A 7.3‐mm S1 iliosacral screw and the trajectory of an S2 iliosacral screw guidewire on a fluoroscopic AP view. (b) A washer‐augmented, partially threaded cannulated S2 iliosacral screw over a guidewire in a fluoroscopic inlet view. (c) S1 and S2 iliosacral screws with a retained anterior plate in a fluoroscopic outlet view.

Because of the revision nature of the procedure and the forces applied during extraction, an additional S2 iliosacral screw (6.5 mm × 70 mm) was inserted for supplementary stabilization (Figure 4).

### 4.5. Neurovascular Safety

The safe screw trajectory was confirmed using the following:

• AP view
• Inlet view
• Outlet view
• Lateral fluoroscopic view

Particular care was taken to avoid the following:

• The L5 nerve root corridor
• The S1 neural foramina

Total operative time was approximately 75 min with blood loss less than 100 mL.

### 4.6. Postoperative Management

The patient had no neurological deficits postoperatively. Bowel and bladder functions were intact. Low molecular weight heparin was administered for DVT prophylaxis. The patient remained nonweight‐bearing for 6 weeks, followed by gradual progression to full weight‐bearing under supervised physiotherapy.

**Figure 5:**
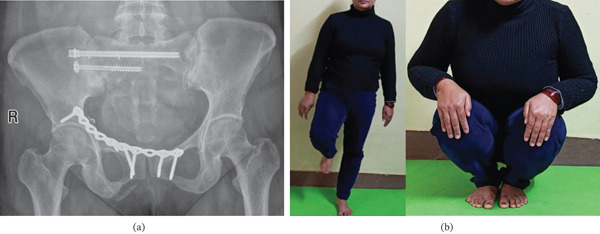
(a) AP pelvis X‐ray at 6‐month follow‐up showing maintained reduction of the anterior and posterior pelvic ring with iliosacral screws and a plate in situ. (b) Clinical image of the patient at 6‐month follow‐up showing excellent function.

At 6‐month follow‐up, the patient reported minimal pain (VAS 1/10) and was able to squat and walk independently without assistive devices (Figure 5b). Radiographs demonstrated a stable implant position without evidence of loosening or displacement (Figure 5a).

## 5. Discussion

Hardware removal in orthopedics can become daunting and challenging, irrespective of the type of case. Removal of broken screws poses its own unique challenges, especially the retrieval of the broken screw shaft [10, 11].

Even in cases of long bones, removal of broken screws is challenging and poses certain risks like iatrogenic fracture and weakening of bones, leading to a stress riser. Several techniques and specialized instruments have been described for the removal of broken orthopedic screws, although their application in the sacroiliac region is limited by anatomy and access constraints. Dedicated broken screw extraction sets, which typically include conical extraction bolts, hollow reamers, and reverse‐threaded devices, are widely used in long bones and can facilitate removal when the proximal fragment is accessible. Reverse‐threaded (counterclockwise) extractors may engage the internal canal of cannulated screws or the fractured core, allowing gradual backing‐out of the retained segment; however, adequate purchase is often difficult in deeply seated sacral fragments [10, 12–16]. Broken iliosacral screws pose a unique challenge owing to their anatomic location, lack of specific extraction devices, a narrow osseous safe zone, proximity to neural elements, and their rarity. This gap in instrumentation, difficult anatomical location, and percutaneous nature of fixation often require the surgeon to improvise with existing tools, sometimes leading to prolonged surgical duration and potential complications [9, 17–20].

Herteleer [11] utilized a navigation‐based technique to remove broken iliosacral screws, providing three‐dimensional guidance to localize the fragment precisely and reduce the risk of neurovascular injury during retrieval in complex pelvic anatomy. In contrast, our approach employed fluoroscopic guidance assisted by a guidewire to safely remove the broken screw. While both techniques aim for accuracy and safe hardware removal, our method elucidates a technique where navigation is not available.

Dafrawy and Osgood [21] reported a nonnavigated, fluoroscopic technique for removing broken iliosacral screws (push screw technique) in two cases. While their approach was similar in terms of fluoroscopic guidance, they explained the use of an extraction device designed specifically for the task. In contrast, we adopted a strategy of using a larger 7.3‐mm screw to push the broken fragment toward the contralateral side, demonstrating the effectiveness of this technique in the absence of dedicated extraction devices. By inserting a larger revision screw along the same trajectory, the retained fragment can be safely advanced toward the contralateral side. Hence, a larger‐diameter screw was required for fixation. Moreover, a biomechanically larger‐diameter screw improves fixation strength by increasing construct stiffness and thread purchase within the sacral cancellous bone, which can be particularly useful in revision situations.

### 5.1. Potential Advantages of the Push‐Through Technique

• Avoids the need for specialized extraction instruments
• Allows removal of deeply embedded fragments
• Preserves the existing osseous corridor
• Permits immediate revision fixation

### 5.2. Potential Limitations of the Push‐Through Technique

• Risk of contralateral cortical breach
• Variation in sacral morphology
• Narrower safe corridor in sacral dysmorphism
• Technical learning curve for surgeons unfamiliar with the technique

Another limitation of this case was the absence of preoperative CT imaging. Although CT scanning provides superior evaluation of sacral morphology, surgical planning in this case relied on conventional radiographs combined with careful intraoperative fluoroscopic guidance.

### 5.3. Potential Complications

• Sacral cortical breach
• Neural injury
• Screw malposition
• Sacral fracture during screw advancement

These risks can be minimized with careful preoperative planning and continuous fluoroscopic monitoring.

A broken iliosacral screw can lead to mechanical instability, impingement, migration into the neural foramina, and most importantly delay in mobilization of the patient [22–24]. Thus, it is important to remove the broken screw and consider refixation to restore stability.

## 6. Conclusion

The push‐through technique described in this report provides a simple and effective method for removing broken sacroiliac screws in the absence of specialized extraction tools or navigation systems. Use of a larger revision screw combined with supplemental S2 fixation can improve biomechanical stability and allow safe management of this challenging complication.

## Funding

No funding was received for this manuscript.

## Ethics Statement

The authors have nothing to report.

## Consent

Written informed consent was obtained from the patient for publication of this case report and accompanying clinical images. A copy of the written consent is available for review by the Editor‐in‐Chief upon request.

## Conflicts of Interest

The authors declare no conflicts of interest.

## Data Availability

The data that support the findings of this study are available from the corresponding author upon reasonable request.
